# Suppression of SOCS3 enhances TRAIL-induced cell growth inhibition through the upregulation of DR4 expression in renal cell carcinoma cells

**DOI:** 10.18632/oncotarget.25851

**Published:** 2018-08-03

**Authors:** Michihiro Yabe, Kei Ishibashi, Akifumi Onagi, Ryo Tanji, Ruriko Honda-Takinami, Tomoyuki Koguchi, Kanako Matsuoka, Seiji Hoshi, Junya Hata, Masao Kataoka, Soichiro Ogawa, Hiroyuki Hiraki, Nobuhiro Haga, Yoshiyuki Kojima

**Affiliations:** ^1^ Department of Urology, Fukushima Medical University School of Medicine, Fukushima, Japan

**Keywords:** renal cell carcinoma, TRAIL-R1/DR4, IFN, SOCS3, tocilizumab

## Abstract

**Background:**

Tumor necrosis factor-related apoptosis-inducing ligand (TRAIL) is a tumor-selective apoptosis inducer that is expressed in natural killer cells, whose cytotoxicity is activated by interferon (IFN). We investigated the effect of suppressor of cytokine signaling (SOCS) 3 on the expression of TRAIL receptors (DR4) and on TRAIL sensitivity in renal cell carcinoma (RCC) cells.

**Methods:**

Vector expression, RNA interference and IL-6 receptor antibody tocilizumab were used to investigate the functional role of SOCS3 in DR4 expression. Immunoprecipitation was employed to detect the biochemical interaction between SOCS3 and DR4. The expression of DR4 induced by combination with IFN-α and tocilizumab was also examined by immunohistochemical staining using mice xenograft model.

**Results:**

DR4 expression was up-regulated by IFN stimulation in RCC cells. 786-O cells were resistant to TRAIL and showed higher SOCS3 expression. ACHN cells showed higher DR4 expression and lower SOCS3 expression. Suppression of SOCS3 up-regulated DR4 expression and enhanced the TRAIL sensitivity in 786-O cells. In ACHN cells, DR4 expression was down-regulated by transfection with pCI-SOCS3, and the cells became resistant to TRAIL. Immunoprecipitation revealed the biochemical interaction between SOCS3 and DR4. A marked increase in IFN-induced DR4 protein expression after tocilizumab treatment was observed by immunohistochemical staining in the tumor from the mice xenograft model.

**Conclusions:**

Our results indicate that IFN and SOCS3 regulate DR4 expression in RCC cells. Combination therapy with IFN-α, tocilizumab and an anti-DR4 agonistic ligand appears to effectively inhibit advanced RCC cell growth.

## INTRODUCTION

Renal cell carcinoma (RCC) is the most prevalent malignancy arising within the kidney [1] and 20% of patients present with advanced diseases which are often difficult to treat [2, 3]. Recently, molecular targeted agents have been used for the treatment of advanced RCC [4, 5] and have shown efficacy against metastatic RCC [5, 6]. However, their effects were still limited and have been shown not to be curative [7].

One group of molecular targeted agents, vascular endothelial growth factor receptor tyrosine kinase inhibitors, is a standard care for advanced RCC [8]. Moreover, nivolumab, a programmed death 1 (PD-1) checkpoint inhibitor, was approved for previously treated patients with advanced RCC, based on the superior overall survival of patients treated with nivolumab versus everolimus [9]. Due to recent advances in treatment options, immunotherapy seems to have a minimal role in the management of advanced RCC. However, the value of immunotherapy for RCC is supported by reports of infrequent complete regression of metastatic disease in response to cytokine therapies, with about 14 percent of cases of metastatic clear-cell renal carcinoma responding to interferon (IFN)–α alone [10]. Thus, IFN–α is still one of the most frequently used immunotherapeutic agents for metastatic or recurrent RCC, especially in cases of lung metastasis.

One of the anti-tumor mechanisms of IFN-α involves the activation of the cytotoxicity of natural killer (NK) cells, of which the cytotoxic molecule tumor necrosis factor-related apoptosis-inducing ligand (TRAIL) plays an important role [11]. TRAIL belongs to the TNF superfamily of proteins and is highly expressed on NK cells [11–13]. TRAIL has received considerable attention because of its selectivity for tumor versus normal tissue and lack of systemic toxicity [14, 15]. TRAIL interacts with four receptors, two death receptors (TRAIL-R1/DR4 and TRAIL-R2/DR5) [16–18] and two decoy receptors (TRAIL-R3/TRID/DcR1 and TRAIL-R4/DcR2) [18–20]. It is suggested that the two death receptors are associated with differences in TRAIL sensitivity between RCC cell lines [21].

We have previously reported that suppressor of cytokine signaling 3 (SOCS3) and interleukin (IL)-6 play important roles in drug resistance in RCC [22, 23]. SOCS3 acts as a negative regulator of IFN-α signaling in the Janus kinase/signal transducer and activator of transcription (JAK/STAT) pathway. In IFN-α-resistant RCC cells, IL-6 induced by IFN stimulation leads to SOCS3 expression, which subsequently inhibits IFN signaling [22, 24]. The silencing of SOCS3 expression or blockade of IL-6 signaling could be a possible strategy to restore sensitivity to IFN-α-resistant RCC cells.

IL-6 is one of the factors associated with poor prognosis of patients with RCC [25, 26]. IL-6 binds to its receptor on tumor cells and activates Janus kinase/signal transducer and activator of transcription (JAK/STAT) pathway. IL-6 activates STAT3 and promote angiogenesis and tumor invasion through VEGF and matrix metalloproteinases expression [27, 28, 29]. Tocilizumab, a humanized antihuman IL-6 receptor (IL-6R) antibody that binds to the IL-6-binding site of human IL-6R and competitively inhibits IL-6 signaling, is available as an approach to the therapeutically effective reagents against inflammatory diseases such as rheumatoid arthritis, juvenile idiopathic arthritis, Castleman's disease, and Crohn's disease [30, 31, 32, 33]. We have reported that tocilizumab in combination with anti-RCC drugs can effectively suppress tumor growth *in vitro* and *in vivo* through repressing activation of STAT3, Akt and mTOR as well as expression of HIF or SOCS3 [22, 23].

As the NK cell activation leading to the anti-tumor effect of TRAIL is induced by IFN, IFN-resistant RCC cells could potentially show resistance to TRAIL. In this study, we showed that the IFN-α-induced expression of TRAIL receptors is dependent on SOCS3 expression. We also show that the suppression of SOCS3, including the blockade of IL-6 signaling, can induce TRAIL sensitivity, thus leading to the inhibition of tumor growth in IFN-α-resistant RCC cells.

## RESULTS

### Sensitivity of RCC cell lines to TRAIL

**Figure 1:**
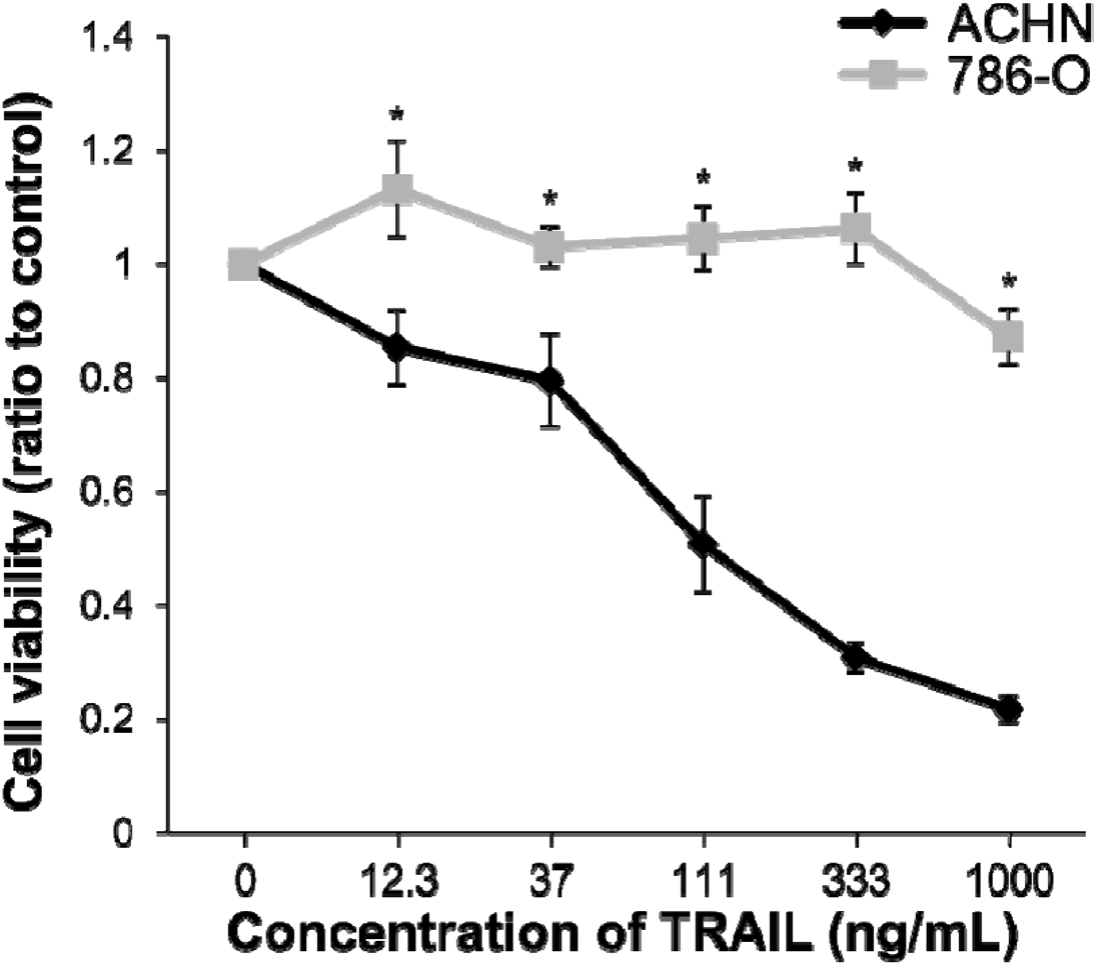
Sensitivity of renal cell carcinoma (RCC) cell lines to tumor necrosis factor-related apoptosis-inducing ligand (TRAIL)-induced cell death ACHN and 786-O cells were treated with recombinant human TRAIL (0-1000 ng/mL) and anti-6X histidine mAb (10 μg/mL). The relative absorbances (mean ± SE) compared with non-treated cells, as a measure of cell viability, obtained from the WST-1 assay are shown. Significant differences were observed at doses of 12.3 ng/mL (*p* < 0.05) and over (*p* < 0.01).

We have previously reported that ACHN cell lines were sensitive and 786-O cell lines were resistant to IFN-α in RCC cell lines [22, 24]. To determine the sensitivity of ACHN and 786-O cells to TRAIL, cell viability assays were carried out. Cell viability in ACHN cells was inhibited by TRAIL treatment in a dose-dependent manner. In contrast, TRAIL did not exert any inhibitory effect on the growth of 786-O cells (Figure 1). The sensitivity of these cell lines to TRAIL was the same as that to IFN-α and was consistent with previously reported results [21]. Cell death was induced in approximately 50% of ACHN cells at a concentration of 111 ng/mL. Thus, the concentration of TRAIL was determined to be 100 ng/mL for the further experiments.

### Gene expression of TRAIL receptors and SOCS3 in RCC cell lines

**Figure 2:**
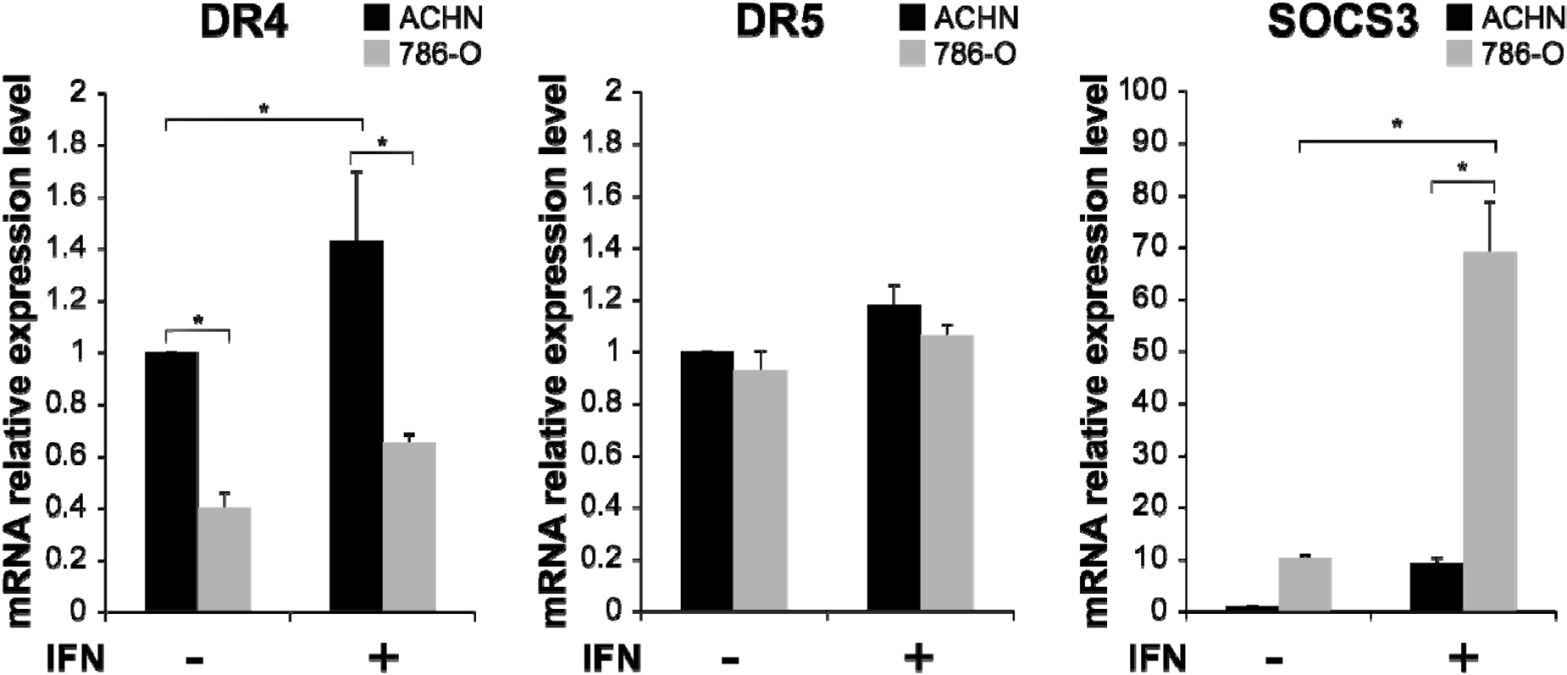
Interferon (IFN)-α-induced mRNA expression of TRAIL-R1/DR4, TRAIL-R2/DR5 and suppressor of cytokine signaling 3 (SOCS3) in RCC cells mRNA expression levels of DR4, DR5 and SOCS3 were quantified by real-time polymerase chain reaction (PCR) in ACHN and 786-O cells. The *y* axis shows the relative mRNA expression level with or without IFN-α treatment (1000 IU/mL) relative to that in non-treated ACHN cells. The results are expressed as the relative mean ratio ± SE of at least three independent determinations. ^*^p < 0.05 for two-tailed paired *t* test compared with ACHN and 786-O cells.

It is known that resistance to TRAIL is in part caused by the reduced expression of DR4 or DR5 [16–20]. When the mRNA expression levels of DR4, DR5 and SOCS3 in RCC cell lines were quantified, DR4 mRNA expression was found to be significantly higher in ACHN cells than in 786-O cells (Figure 2, *p* < 0.001). After IFN-α stimulation, the DR4 mRNA expression level increased in both ACHN and 786-O cells compared with that in pretreated cells, with the difference in the ACHN cells, but not that in 786-O cells, being significant (*p* = 0.044). In contrast, the SOCS3 mRNA expression level was significantly higher in 786-O cells than in ACHN cells (*p* < 0.001), and these levels were significantly increased by IFN-α stimulation (*p* < 0.001). The DR5 mRNA expression level was higher in ACHN cells than in 786-O cells, but no significant differences were observed. These results suggested that the difference in TRAIL sensitivity was regulated not by DR5 but by DR4 expression in those cells. 786-O cells were resistant to TRAIL despite no differences being observed in DR5 mRNA expression level after treatment with IFN. Thus, we decided to evaluate the relationship between DR4 and SOCS3 in this study.

### Correlation between DR4 and SOCS3

**Figure 3:**
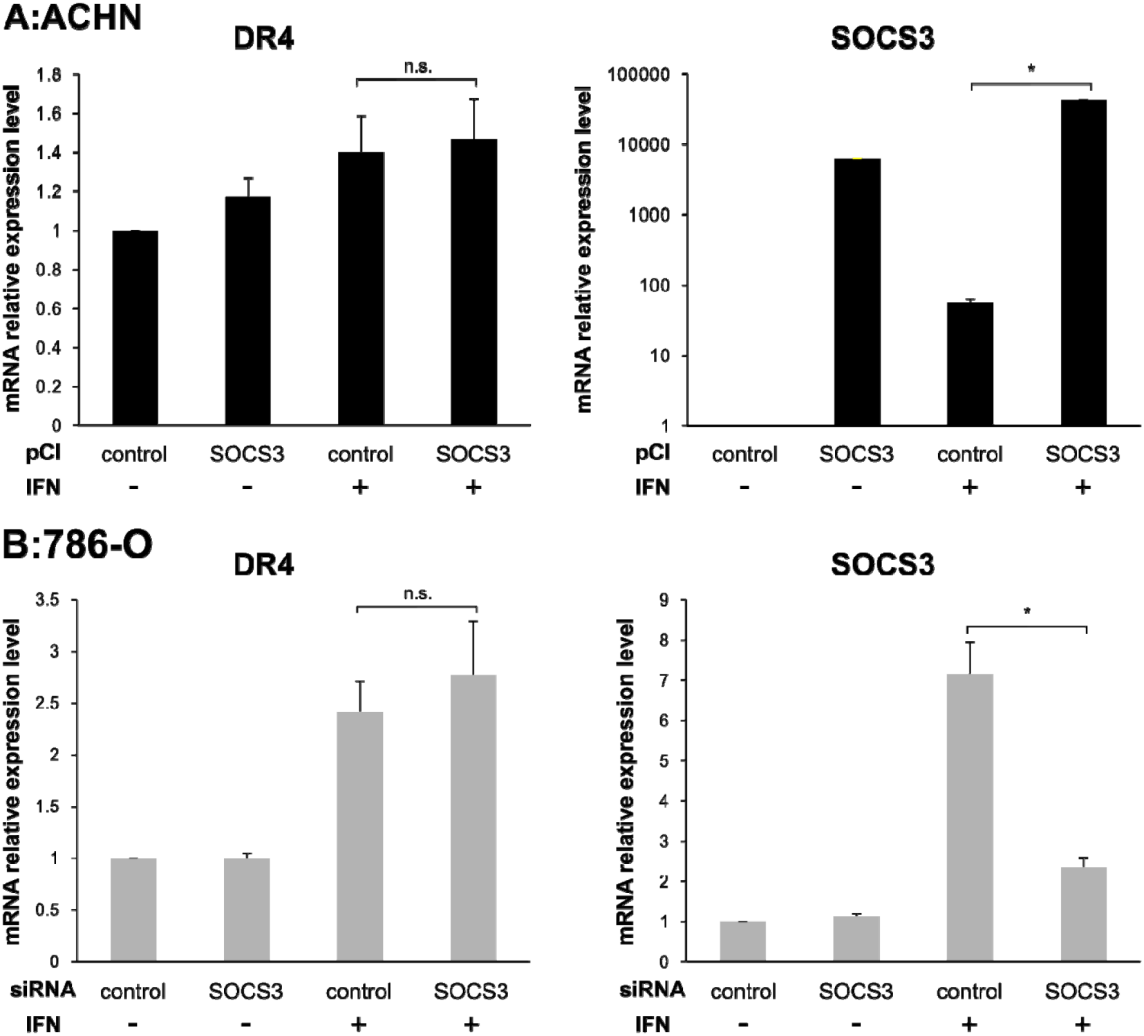
Effect of pCI-SOCS3 vector or SOCS3 siRNA transfection on DR4 mRNA expression mRNA expression levels of DR4, DR5 and SOCS3 were quantified by real-time PCR. DR4 mRNA expression was increased by IFN-α stimulation but not by SOCS3. The *y* axis shows the relative mRNA expression level. The results are expressed as the relative mean ratio ± SE of at least three independent determinations. ^*^p < 0.05 for two-tailed paired *t* test compared with control.

**Figure 4:**
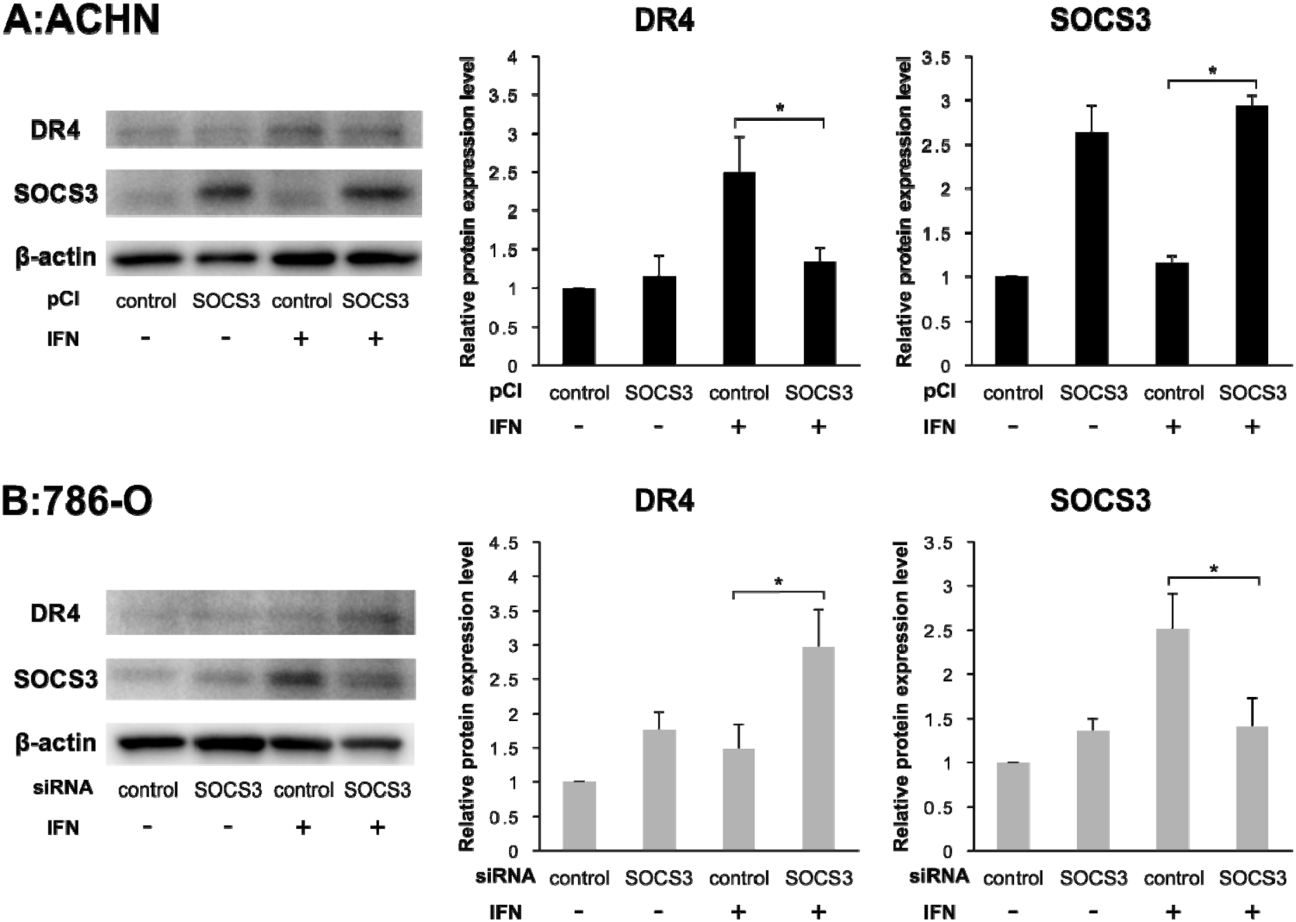
DR4 protein expression depends on SOCS3 Individual bands of the Western blotting analysis were quantified using Image Lab 3.0 software and compared with protein expression levels. **(A)** Effect of pCI-SOCS3 on ACHN cells. Overexpression of SOCS3 suppressed the DR4 expression by IFN in ACHN cells. **(B)** Effect of SOCS3-siRNA on 786-O cells. DR4 protein expression was dependent on SOCS3 expression. The results are expressed as the relative mean ratio ± SE of at least three independent determinations. ^*^p < 0.05 for two-tailed paired *t* test compared with control.

To evaluate the correlation between DR4 and SOCS3, we quantified the expression levels of DR4 and SOCS3 after transfection of pCI-SOCS3 into ACHN cells and SOCS3-siRNA into 786-O cells. The DR4 mRNA expression level was increased after IFN-α stimulation in both ACHN cells and 786-O cells as mentioned above but was not influenced by SOCS3 expression (Figure 3). However, in ACHN cells, Western blotting analysis showed that the DR4 protein expression level was increased after IFN-α stimulation in the control cells, whereas it was not increased in pCI-SOCS3-transfected cells (Figure 4A, *p* = 0.011). In 786-O cells, on the other hand, the DR4 protein expression level was significantly increased after IFN-α stimulation in SOCS3-siRNA-transfected cells (*p* = 0.009), whereas it was not increased in the control cells (Figure 4B).

### Influence of SOCS3 on sensitivity to IFN and TRAIL

**Figure 5:**
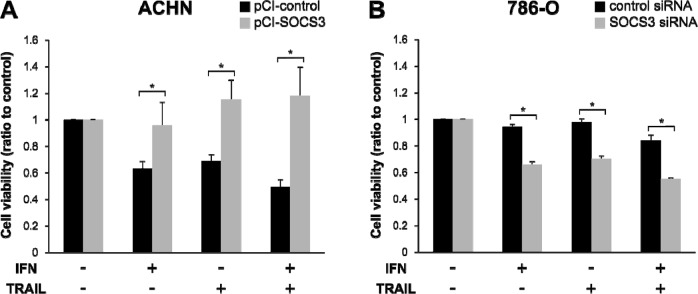
Reduced cell viability in response to TRAIL depends on SOCS3 expression Cell viability after treatment with IFN-α and/or TRAIL were compared with non-treated cells. **(A)** ACHN cells seeded on a culture plate were transfected with an empty vector or pCI-SOCS3 vector. **(B)** 786-O cells were transfected with negative control siRNA or SOCS3 siRNA. Cell viability after TRAIL treatment changed in a SOCS3-dependent manner. ^*^p < 0.05 for two-tailed paired *t* test compared with control.

To evaluate the influence of SOCS3 on sensitivity to IFN-α and TRAIL, we carried out cell-viability studies after transfection of pCI-SOCS3 into ACHN cells and SOCS3-siRNA into 786-O cells. The ACHN cells became resistant to IFN-α and TRAIL after pCI-SOCS3 transfection (Figure 5A, *p*<0.001). In 786-O cells, on the other hand, the growth of SOCS3-siRNA-transfected cells was significantly inhibited by treatment with both IFN-α and TRAIL (Figure 5B, *p* < 0.001).

### Binding of SOCS3 to DR4

**Figure 6:**
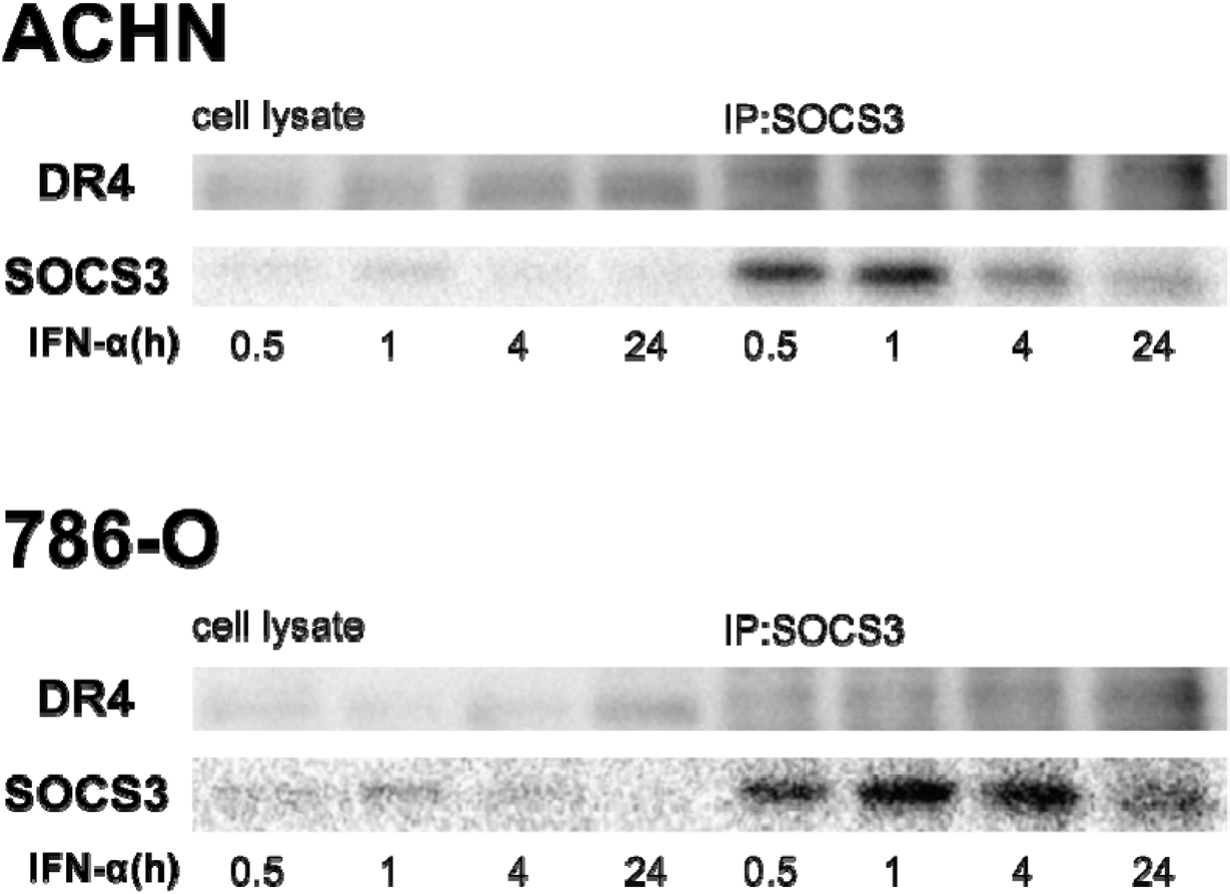
SOCS3 binds to DR4 protein We investigated the biochemical interaction between SOCS-3 and DR-4 by co-immunoprecipitation. Cells were treated with 1000 IU/mL of IFN-α for different time periods. Cells were then dissolved in lysis buffer and proteins were immunoprecipitated with anti-SOCS3 antibody. Cell lysates and immunoprecipitants were applied to SDS-PAGE and Western blotting analyses were carried out with anti-SOCS3 and anti-DR4 antibodies.

SOCS box-containing proteins form complexes with other proteins, thus tagging them for ubiquitination and protein degradation. Hence, we investigated the biochemical interaction between SOCS3 and DR4 by co-immunoprecipitation in ACHN and 786-O cells treated with IFN-α. Protein complexes were precipitated with anti-SOCS3 antibody. Western blot analysis with anti-DR4 antibodies showed that SOCS3 bound to DR4 (Figure 6).

### Changes in DR4 expression and sensitivity to TRAIL under an IL-6 signaling blockade

**Figure 7:**
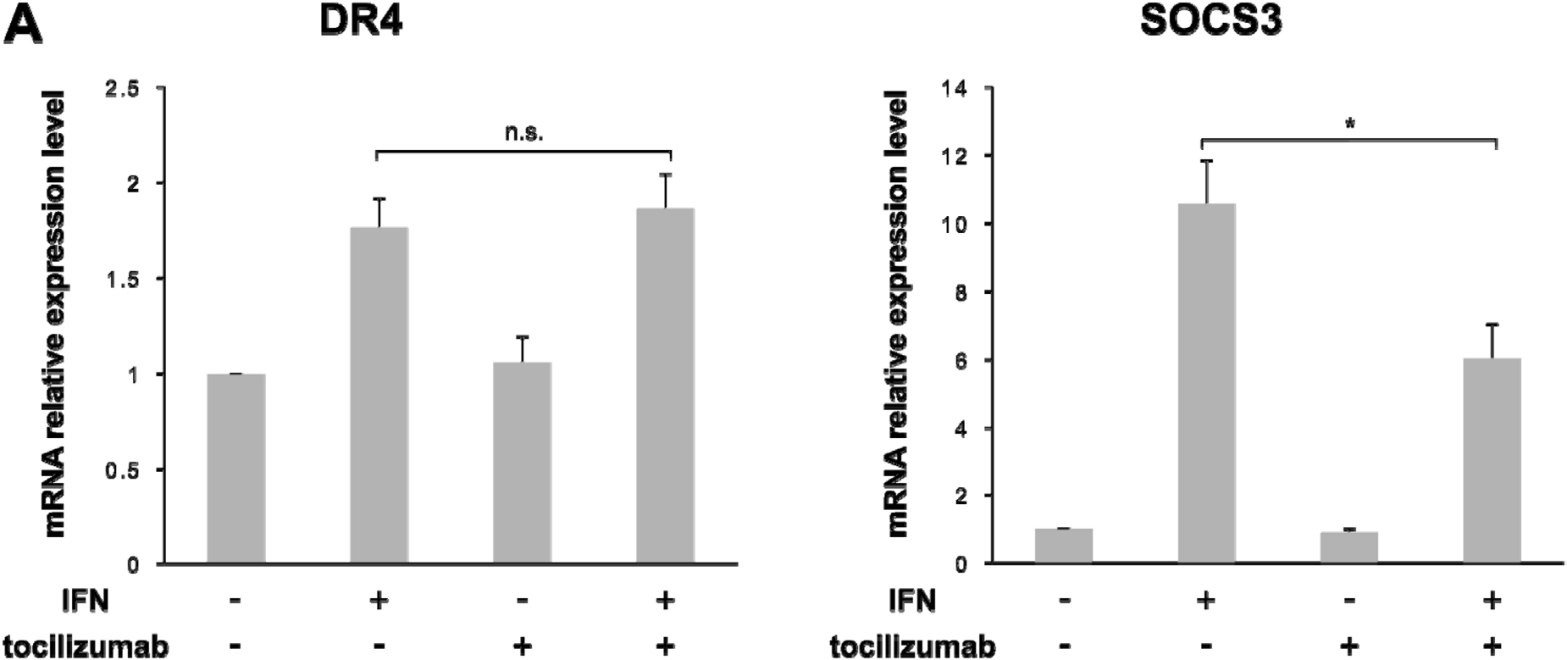

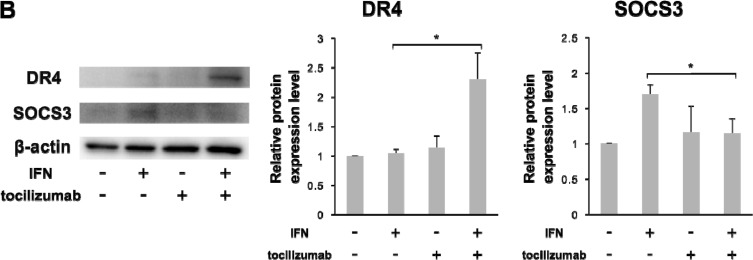

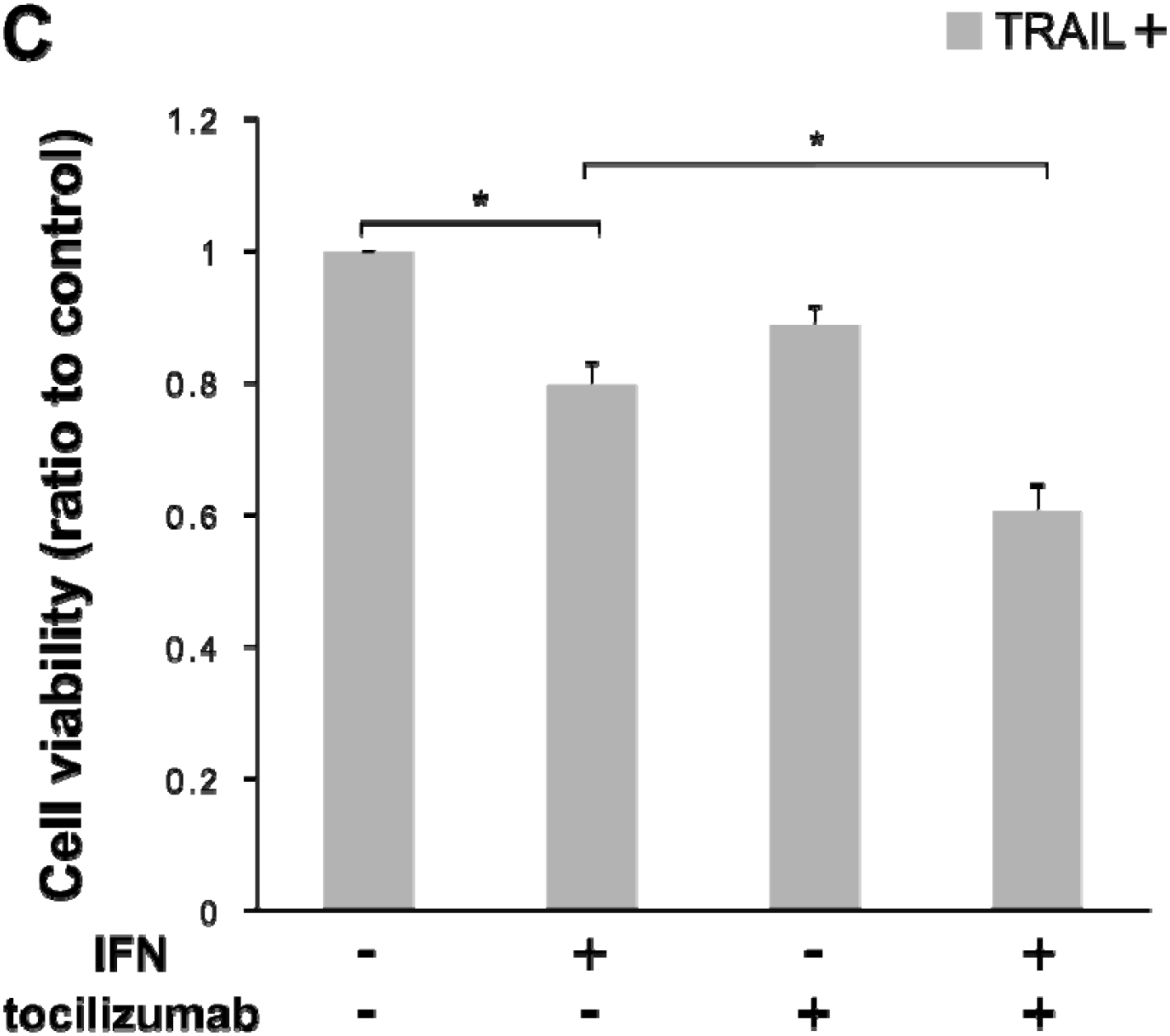
Combination of IFN-α and tocilizumab lead the inhibition of cell viability in response to TRAIL Antihuman IL-6 receptor antibody, tocilizumab, was used for the inhibition of IL-6 signaling 786-O cells were treated with IFN-α and/or tocilizumab (50 μg/mL). **(A)** Effect of IFN-α and tocilizumab on mRNA expression of DR4 and SOCS3. **(B)** Effect of IFN-α and tocilizumab on the protein expression of DR4 and SOCS3. **(C)** 786-O cells were treated with IFN-α, tocilizumab and/or TRAIL. IFN-α treatment with SOCS3 suppression increased DR4 protein expression. Combined use of IFN-α, tocilizumab and TRAIL markedly inhibited cell growth. The results are expressed as the relative mean ratio ± SE of at least three independent determinations. ^*^p < 0.05 for two-tailed paired *t* test compared with control.

We examined the changes in DR4 expression and TRAIL sensitivity under a blockade of IL-6 signaling in 786-O cells. The up-regulation of SOCS3 mRNA expression level by IFN-α stimulation was significantly decreased by the combined use of tocilizumab (*p* = 0.012), but the DR4 mRNA expression level was not influenced by tocilizumab (Figure 7A). However, in terms of protein expression, tocilizumab up-regulated DR4 expression (*p* = 0.002) after IFN-α stimulation but did not induce SOCS3 expression (*p* = 0.010) (Figure 7B). In the cell-proliferative studies, we examined the changes in TRAIL sensitivity by the administration of IFN-α and/or tocilizumab. TRAIL exerted a growth inhibitory effect after IFN-α administration (*p* < 0.001). Moreover, the combined use of tocilizumab markedly increased its effect (*p* < 0.001). Tocilizumab alone also exerted a growth inhibitory effect, but the difference was not significant (Figure 7C).

### Effect of the combined use of IFN-α and tocilizumab on DR4 expression *in vivo*

**Figure 8:**
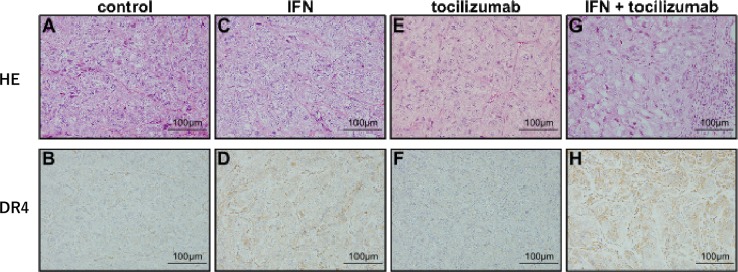
Immunohistochemical staining of tumor xenografts (HE: A, C, E and G. DR4: B, D, F and H.) Histological features and DR4 expression of tumors at 50 days after 786-O cells inoculation. **(A** and **B)** Control mice. **(C** and **D)** Administration of IFN-α. **(E** and **F)** Administration of tocilizumab. **(G** and **H)** Combination of IFN-α and tocilizumab. Tocilizumab administration alone produced no remarkable morphological changes in HE staining or DR4 protein expression. DR4 protein was expressed at a higher level after IFN-α stimulation in comparison with the control, and the combination of IFN-α and tocilizumab markedly increased its expression.

Tumors from the control mice displayed a clear cell type specific to renal cell carcinomas (Figure 8A). Immunohistochemical examination of DR4 confirmed the presence of little or negligible DR4 protein expression in the tumor cells of non-treated mice (Figure 8B). Tumor sections obtained after IFN-α treatment showed no remarkable change in HE staining (Figure 8C); however, DR4 protein expression was observed in the tumor (Figure 8D). No remarkable changes due to the administration of tocilizumab alone were observed (Figure 8E and 8F). Lymphocyte infiltration and focal fibrosis were observed in the tumors from mice receiving a combination of IFN-α and tocilizumab (Figure 8G). A marked increase in IFN-induced DR4 protein expression after tocilizumab treatment was confirmed (Figure 8H).

## DISCUSSION

The present study demonstrates that a combination of IFN and an anti-IL-6 receptor antibody could up-regulate TRAIL receptor DR4 in RCC cells. There are multiple regulatory components in the TRAIL/TRAIL receptor system. In this study, we have focused on the regulation of SOCS3 induction in RCC cells as one of the TRAIL regulatory systems.

We have previously reported that SOCS3 plays an important role in IFN-α resistance in RCC. When IFN-α binds to its receptor, the JAK/STAT pathway is activated and STAT1 is subsequently phosphorylated and acts as an anti-tumor activator. In 786-O cells, which are resistant to IFN, IL-6 is induced by IFN-α stimulation, leading to SOCS3 expression. SOCS3 then inhibits STAT1, which subsequently leads to IFN-α resistance [22, 24]. On the other hand, IFN and STAT1 activate NK cell cytotoxicity [34–36]. As TRAIL, an inducer of apoptosis, is expressed on NK cells, we hypothesized that IFN-resistant RCC cells were also resistant to TRAIL, the receptors of which are regulated by SOCS3.

In this study, we demonstrated that IFN and SOCS3 regulated the expression of TRAIL receptors DR4 as well as TRAIL sensitivity. There was a significant difference in DR4 expression between ACHN and 786-O cells. This suggests that TRAIL sensitivity was mainly regulated by DR4 expression. In our study, DR4 expression was up-regulated by IFN-α stimulation and was inhibited by SOCS3. Cell-proliferative assays using recombinant TRAIL also demonstrated that SOCS3 was one of regulators of TRAIL-induced cell death. In 786-O cells, the DR4 mRNA expression level was increased by IFN-α; however, DR4 expression was not changed. DR4 protein expression was induced by IFN-α under SOCS3 suppression by siRNA. A plausible explanation for our findings is that DR4 was degraded by SOCS3. SOCS3 contains SOCS-box and can form a complex that recognizes activated signaling proteins for ubiquitination and proteasome-dependent degradation. SOCS3 acts as an adapter that brings ubiquitin ligases into the vicinity of activated signaling proteins, causing their ubiquitination [37, 38]. The results of our immunoprecipitation experiment showed that DR4 was a binding partner of SOCS3.

The humanized antihuman IL-6 receptor antibody, tocilizumab, can also suppress SOCS3 expression. As expected, in 786-O cells, the combination of IFN-α and tocilizumab induced DR4 protein expression with decreased SOSC3 expression. Thus, the combined use of IFN-α and tocilizumab markedly enhanced the growth inhibitory effect of TRAIL, although use of IFN-α alone also led to a TRAIL-induced growth inhibitory effect to a certain degree. Our *in vivo* study confirmed that DR4 expression in mice treated with IFN-α alone was higher than that in control mice, but lower than that in mice treated by a combination of IFN-α and tocilizumab. IFN-α treatment induced SOCS3 expression in 786-O cells as previously reported [24]; thus, our results indicate that DR4 degradation by IFN-induced SOCS3 expression was insufficient. However, as the combined treatment with IFN-α and tocilizumab induced higher DR4 expression compared with that of IFN-α treatment alone, it is suggested that IL-6-induced SOCS3 has, at least in part, a role in DR4 degradation in RCC cells. In addition to the tocilizumab, epigenetic modifiers could be a well candidate for controlling renal cancer cell proliferation and immune regulators [39]. Many epigenetic alterations have been identified in urologic cancers including histone modifications and DNA methylation changes. And the resistance to immunomodulatory therapy with interferons in RCC can occur via promoter hypermethylation and silencing of interferon response genes [40]. These changes are reversible and treatment of renal cancer cell lines with 5-Aza-2′-deoxycitidine increased expression of interferon response genes and restored interferon induced apoptosis [40]. It is also reported that histone deacetylase inhibitors (HDACi) could reduce progression in mice RCC syngeneic transplanted model [41]. Clear cell RCC is associated with inactivation of the von-Hippel Lindau (VHL) gene by either genetic and epigenetic factors [42, 43]. It has been reported that von Hippel-Lindau (VHL) gene regulates the SOCS expression [44]. It will also be interesting to investigate the effect of HDACi on SOCS3 expression in RCC cells.

Recently, multiple studies have documented the efficacy of mapatumumab, a human agonistic monoclonal antibody against DR4 [45–47]; however, the use of mapatumumab has not shown clinical benefits in clinical trials [48, 49]. In these studies, mapatumumab was adjunctively used with other anti-cancer or molecular targeted agents. We have recently reported that molecular targeted agents induce IL-6 in RCC cells [23]. Although a major limitation of our study is that we could not use mapatumumab in our setting, it is possible that combination therapy with mapatumumab and molecular targeted agents against RCC requires the suppression of IL-6 signaling for better effects, as IL-6 signaling induces SOCS3 expression.

In conclusion, the present study showed that SOCS3 was one of the regulators of TRAIL sensitivity in RCC. Suppression of SOCS3 expression up-regulated the DR4 expression induced by IFN-α stimulation both *in vitro* and *in vivo*, and enhanced TRAIL sensitivity in RCC cell lines. Our findings suggested that combination therapy using IFN-α with a SOCS3 suppressor and TRAIL could afford an attractive candidate for the treatment of advanced RCC.

## MATERIALS AND METHODS

### Cell lines/ recombinant human TRAIL/ IFN-α/ tocilizumab

The human RCC cell lines ACHN and 786-O were obtained from the American Tissue Culture Collection (ATCC). ACHN and 786-O were cultured at 37°C in 5% CO_2_ using MEM with 0.1mmol/L non-essential amino acids, plus 10% fetal bovine serum for ACHN cells and RPMI 1640 with 10% fetal bovine serum for 786-O cells. Recombinant human (rh) TRAIL and anti-6X histidine mAb were purchased from R&D Systems, Inc. (Minneapolis, MN). Natural type IFN-α (Sumiferon: Dainippon Sumitomo Pharma Co., Japan) was used in this experiment. IFN-α was applied at a dose of 1000 IU/mL as described previously [24]. Tocilizumab, a humanized antihuman IL-6R antibody, was purchased from Chugai Pharmaceutical Co. (Tokyo, Japan). The optimum concentration of tocilizumab was determined to be 50 μg/mL from a pharmacokinetic analysis of rheumatoid arthritis patients as described previously [22, 50].

### cDNA construction and real-time quantitative PCR

cDNA construction was performed using SuperPrep Cell Lysis & RT Kit for qPCR (Toyobo Co., Japan) according to the manufacturer's instructions. TaqMan PCR reagents for DR4 (Hs00269492), DR5 (Hs00366278) and SOCS3 (Hs02330328) were purchased from ABI (Applied Biosystems, CA). Quantitative real-time PCR was carried out using the TaqMan Master Mix Reagents Kit protocol on a StepOne real-time PCR System (Applied Biosystems, CA). The data were standardized against β-actin gene expression using TaqMan β-actin control reagent (Applied Biosystems, CA).

### Short Silencer RNA (siRNA) and transfection

The designed siRNA for SOCS3 and the negative control, which were designed as described previously [24], were also used in this study. For siRNA transfection, Lipofectamine RNAi MAX (Invitrogen, CA) was used according to the manufacturer's protocol. The transfected cells were kept at 37°C in a 5% CO_2_ incubator for 24 h until treatment.

### pCIneo-SOCS3 and transfection

The construct pCIneo-SOCS3 was made by subcloning the human SOCS3 cDNA amplified from 786-O cells into the pCIneo expression vector (Promega, WI) as described previously [24]. The empty vector was used as a negative control. For pCIneo-SOCS3 vector and empty vector transfection, Lipofectamine LTX (Invitrogen, CA) was used according to the manufacturer's protocol. The transfected cells were kept at 37°C in a 5% CO_2_ incubator for 24 h until treatment.

### Cell-viability assay

For the determination of cellular proliferation and viability, water soluble Tetrazolium (WST)-1 assays were carried out in 96-well plates using a Cell Proliferation Reagent (Roche Applied Science, IN) according to the manufacturer's protocol. Briefly, 24 h after the incubation of cells with TRAIL, IFN-α and/or tocilizumab, WST-1 reagent was added to each well, which were then incubated for 1 h at 37°C. Spectrophotometrical absorbance of the samples was measured using a microplate reader (VARIOSKAN FLASH, Thermo Scientific, IL), and compared against that of non-treated cells.

### Western blotting analysis

Protein from the IFN- and/or tocilizumab-treated cells was extracted and applied to SDS-PAGE. DR4 (Santa Cruz Biotechnology, Inc., TX) and SOCS3 (Immuno-Biological Laboratories Co., Japan) were used as primary antibodies. Anti β-actin antibody (SIGMA, MO) was used as an internal control. Protein bands were visualized using SuperSignal West Dura Extended Duration Substrate (Thermo Scientific, IL), and imaged with the ChemiDoc XRS plus system (BIO-RAD, CA). Individual bands were quantified with Image Lab 3.0 software (BIO-RAD, CA), and normalized against the control value.

### Immunoprecipitation

For immunoprecipitation, a PureProteome Protein Magnetic Bead system (Merckmillipore Co., Germany) was used according to manufacturer's protocol. SOCS3 antibody (Santa Cruz Biotechnology, Inc., TX) and Protein G were used to make the antibody-coated beads. Western blotting analysis was then carried out as described above. EasyBlot (GeneTex, CA) was used as a secondary antibody, according to the manufacturer's protocol.

### Tumor xenografts

All animal studies were conducted in compliance with Japanese regulations for animal use and approval for these studies was obtained from the Committee on Animal Research of Fukushima Medical University. Six-week-old female BALB/C nu/nu nude mice (CLEA Japan, Inc.) were inoculated subcutaneously (s. c.) in the flank with 20 million 786-O cells. The tumor-bearing mice were separated into four groups. One group received an intraperitoneal (i. p.) injection of 100 μg antihuman IL-6R antibody tocilizumab three times a week and 1000 IU of IFN-α daily. The other groups received IFN-α only, tocilizumab only or phosphate-buffered saline (PBS; non-treated control group), respectively. After treatment for 50 days, the tumors were removed and used for morphological analysis.

### Morphological and immunohistochemical examinations

Paraffin-embedded sections of the tumors from the mouse xenograft models were prepared and stained with hematoxylin and eosin (HE). To detect DR4, tumor sections were stained with a polyclonal rabbit anti-TNFRSF10A/DR4 antibody (LifeSpan BioScience, Inc., WA). Staining was detected using a Streptavidin-Biotin Kit (Nichirei, Trappes, France) according to the manufacturer's protocol.

### Statistical analysis

Determination of cell proliferation, mRNA expression level and Western blotting analysis was repeated at least three times independently, and the results were expressed as the mean ± SE. Analyses were performed with SPSS Statistics 21 software (IBM Japan, Tokyo, Japan).

## Abbreviations

IL-6: interleukin 6
IL-6R: interleukin 6 receptor
JAK/STAT: janus kinase/signal transducer and activator of transcription
RCC: renal cell carcinoma
SOCS: suppressor of cytokine signaling
TRAIL: tumor necrosis factor-related apoptosis-inducing ligand.
